# The Effect of Vinpocetine on Human Cytochrome P450 Isoenzymes by Using a Cocktail Method

**DOI:** 10.1155/2016/5017135

**Published:** 2016-02-24

**Authors:** Lingti Kong, Chunli Song, Linhu Ye, Daohua Guo, Meiling Yu, Rong Xing

**Affiliations:** ^1^Department of Pharmacy, The First Affiliated Hospital of Bengbu Medical College, Bengbu 233000, China; ^2^Institute of Medicinal Plant Development, Chinese Academy of Medical Sciences and Peking Union Medical College, Beijing 100193, China; ^3^Department of Pharmacy, The First People's Hospital of Bijie, Bijie 551700, China

## Abstract

Vinpocetine is a derivative of the alkaloid vincamine, which had been prescribed for chronic cerebral vascular ischemia and acute ischemic stroke or used as a dietary supplement for its several different mechanisms of biological activities. However, information on the cytochrome P450 (CYP) enzyme-mediated drug metabolism has not been previously studied. The present study was performed to investigate the effects of vinpocetine on CYPs activity, and cocktail method was used, respectively. To evaluate the effects of vinpocetine on the activity of human CYP3A4, CYP2C9, CYP2C19, CYP2D6, and CYP2E1, human liver microsomes were utilized to incubate with the mixed CYPs probe substrates and the target components. The results indicate that vinpocetine exhibited weak inhibitory effect on the CYP2C9, where the IC_50_ value is 68.96 *μ*M, whereas the IC_50_ values for CYP3A4, CYP2C19, CYP2D6, and CYP2E1 were all over range of 100 *μ*M, which showed that vinpocetine had no apparent inhibitory effects on these CYPs. In conclusion, the results indicated that drugs metabolized by CYP2C9 coadministrated with vinpocetine may require attention or dose adjustment.

## 1. Introduction

**Figure 1:**
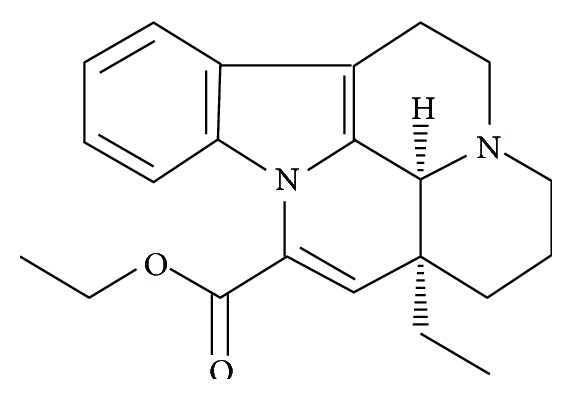
Chemical structures of vinpocetine.

Vinpocetine, ethyl (3*α*,16*α*)-eburnamenine-14-carboxylate (Figure 1), which is a derivative of the alkaloid vincamine had found several different mechanisms of biological activities, including neuroprotective [1], anti-inflammatory [2, 3], antinociceptive [4], and antiseizure activities [5]. Currently, vinpocetine is primarily prescribed for chronic cerebral vascular ischemia and acute ischemic stroke in China, Germany, Japan, Hungary, Poland, Russia, and other countries [6]. In addition, more than 300 brands of dietary supplements, which are regulated as food and hence sold directly to consumers, labelled as containing vinpocetine, are available for sale in the United States [7]. However, vinpocetine has displayed adverse effects including conditions such as facial flushing, headaches, and drop of blood pressure [7].

Vinpocetine readily enters the bloodstream from the stomach and gastrointestinal tract and consequently passes the blood-brain barrier. Vinpocetine was metabolized exclusively in the liver of dogs and humans, whereas in rats extrahepatic metabolism seems to be important [8, 9]; apovincaminic acid is the main hydrolysis metabolite of vinpocetine and is eliminated from the body through the kidneys [10].

Adverse drug reaction (ADR) is one of the major causes of morbidity and mortality occurring in clinical care every year [11, 12] and 31.5% of drug-drug interactions (DDIs) potentially contributed to ADRs [13]. DDIs, which can be classified into pharmacokinetic and pharmacodynamic, might be associated with serious or even fatal adverse events, or can lead to reducing therapeutic effects of either drug [14], are common in the elderly due to polytherapy. Polytherapy increases the complexity of therapeutic management and thereby the risk of clinically relevant DDIs [15]. Pharmacokinetic interactions arise when absorption, distribution, metabolism, or elimination of the involved drugs is altered, leading to changes in the amount and duration of drug availability at receptor sites. More precisely, the most common DDI of pharmacokinetics may be understood in terms of metabolic alterations, primarily associated with changes in the activity of cytochrome P450 (CYP) enzymes [16].

Although there have been some investigations of DDI of vinpocetine [9, 16, 17], information on the CYP enzyme-mediated drug metabolism has not been previously studied. Owing to the fact that the use of* in vitro* data to predict the inhibition potential of a drug is wonderful with simple, convenient, and high throughput [18], the major objective of the present study was to investigate the effects of vinpocetine on the CYP3A4, CYP2C9, CYP2C19, CYP2D6, and CYP2E1 enzymes, which are primarily involved in drug metabolism, and then to predict any DDIs when vinpocetine is coadministered with other drugs metabolized by CYPs.

## 2. Material and Methods

### 2.1. Chemicals and Reagents

Chlorzoxazone, dextromethorphan, tolbutamide, testosterone, omeprazole, 6-hydroxychlorzoxazone, dextrorphan, and 5-hydroxyomeprazole were purchased from Sigma-Aldrich Company (St. Louis, USA). 6*β*-Hydroxytestosterone and 4-hydroxytolbutamide were purchased from Toronto Research Chemicals Inc. (Toronto, Canada). *β*-Nicotinamide adenine dinucleotide phosphate (NADPH) was purchased from Roche (Roche, Switzerland). Vinpocetine, propranolol, and gliclazide were obtained from the National Institutes for Food and Drug Control (Beijing, China). Acetonitrile of high-performance liquid chromatography grade was obtained from Fisher Co. Ltd. (Waltham, MA, USA). Milli-Q (Milford, MA, USA) water was used throughout the experiments.

### 2.2. Human Liver Microsomes

A pooled sample of human liver microsomes was obtained from BD Gentest Corporation (BD Gentest*™*, Woburn, USA). The microsomes were frozen and stored at –80°C until used.

### 2.3. Microsomal Incubations

To evaluate the effects of vinpocetine on the activity of human CYP3A4, CYP2C9, CYP2C19, CYP2D6, and CYP2E1, human liver microsomes were used to incubate with the mixed CYPs probe substrates (dextromethorphan/testosterone/omeprazole/chlorzoxazone/tolbutamide) and vinpocetine at different concentrations [19].

Briefly, each mixture (100 *μ*L) contained 100 mM phosphate buffer (pH 7.4), 3.3 mM MgCl_2_, 1 mM NADPH, 0.3 mg/mL microsomal protein, five probe substrates, and the tested sample or blank solvent (control). The final concentrations of the probe substrates were 100 mM for tolbutamide, 5 mM for dextromethorphan, and all 50 mM for omeprazole, chlorzoxazone, and testosterone, and the final concentrations of the vinpocetine in the incubation were at serial concentrations of 0.1, 0.3, 1.0, 3.0, 10.0, 30.0, and 100 *μ*M, respectively. The reaction mixture, which includes microsomes, substrates, and the vinpocetine in a final volume of 100 *μ*L, was preincubated for 10 min at 37°C in a shaking water bath, and the reaction was initiated through the addition of NADPH and terminated with 100 *μ*L of ice-cold acetonitrile containing 1 *μ*g/mL propranolol (internal standard for positive mode) and 1 *μ*g/mL gliclazide (internal standard for negative mode) after 30 min incubation. After vortexing, the mixtures were centrifuged at 15,000 g for 10 min at 4°C. Two 10 *μ*L aliquots of the supernatant were injected directly into the LC-MS/MS system for the determination of the produced metabolites (dextrorphan/6*β*-hydroxytestosterone/5-hydroxyomeprazole/6-hydroxychlorzoxazone/4-hydroxytolbutamide).

### 2.4. Chromatographic Conditions

The metabolites of five CYPs probe substrates in all samples were identified by using our previously developed LC-MS/MS method [20]. In brief, the produced metabolites in the incubation mixtures were performed with an Agilent 1200 HPLC (Palo Alto, CA, USA) equipped with a quaternary pump, an autosampler, a thermostated column compartment, and an Applied Biosystem 3200 Q-Trap (Foster City, CA, USA) equipped with an electrospray ion source. The samples were separated on an Agilent RP-C_18_ column (2.1 × 50 mm, 3.5 *μ*M) with the column temperature at 40°C. The mobile phase consisted of 0.1% formic acid in water (A) and in acetonitrile (B) with following gradient elution at a flow rate of 0.4 mL/min: 0–0.1 min, 5–90% A; 0.1–4.1 min, 90% A; 4.1–7.0 min, 90–5% A. The ion spray voltage was operated separately in the positive ion mode at 5500 V and the negative ion mode at −4000 V, respectively. The operating conditions were the following: ion source temperature, 400°C; curtain gas, 20 psi; ion source gas 1, 60 psi; ion source gas 2, 60 psi. The quantification was performed by multiple reaction monitoring (MRM) of the molecular ion and the related product ion for each metabolite, ESI^+^:* m*/*z* 258.1 → 157.2 for dextrorphan,* m*/*z* 305.1 → 269.3 for 6*β*-hydroxytestosterone,* m*/*z* 362.3 → 214.2 for 5-hydroxyomeprazole, and* m*/*z* 261.3 → 116.1 for propranolol; ESI^−^:* m*/*z* 183.8 → 119.9 for 6-hydroxychlorzoxazone,* m*/*z* 285.4 → 185.6 for 4-hydroxytolbutamide, and* m*/*z* 322.4 → 170.2 for gliclazide.

### 2.5. Data Analysis

The data acquisition and peak integration were performed by utilizing analyst software (Version 1.4.2). The ratios were plotted as a percentage of the relevant control for each metabolic reaction, and the half maximal inhibitory concentration (IC_50_) for each CYP isozyme was calculated using a nonlinear regression analysis program in GraphPad Prism 5.0 (GraphPad Software 5.0).

All the experiments were done in triplicate, and the data were expressed as mean ± SD.

## 3. Results

### 3.1. Method Validation

The concentrations of dextrorphan, 6*β*-hydroxytestosterone, 5-hydroxyomeprazole, 6-hydroxychlorzoxazone, and 4-hydroxytolbutamide were determined by a sensitive and simple UPLC-MS/MS method [20]. The calibration curves between the peak area ratios of metabolites/IS against the metabolites concentrations displayed good linearity with correlation coefficients all higher than 0.99, with ranges from 1.50 to 1500.00, 10.00 to 4000.00, and 2.50 to 2000.00 *μ*g/mL for dextrorphan, 6*β*-hydroxytestosterone, and other metabolites, respectively. The limits of detection (LOD) for dextrorphan, 6*β*-hydroxytestosterone, 5-hydroxyomeprazole, 6-hydroxychlorzoxazone, and 4-hydroxytolbutamide were 0.50, 5.00, 1.00, 1.00, and 0.50 ng/mL, respectively. The method showed excellent reproducibility with intraday and interday precision less than 12.00%, and the accuracy ranged from 88.96% to 114.73%. The matrix effects were more than 88.59% or less than 112.34%.

### 3.2. Inhibitory Effects of Vinpocetine on P450 Activity

**Figure 2:**
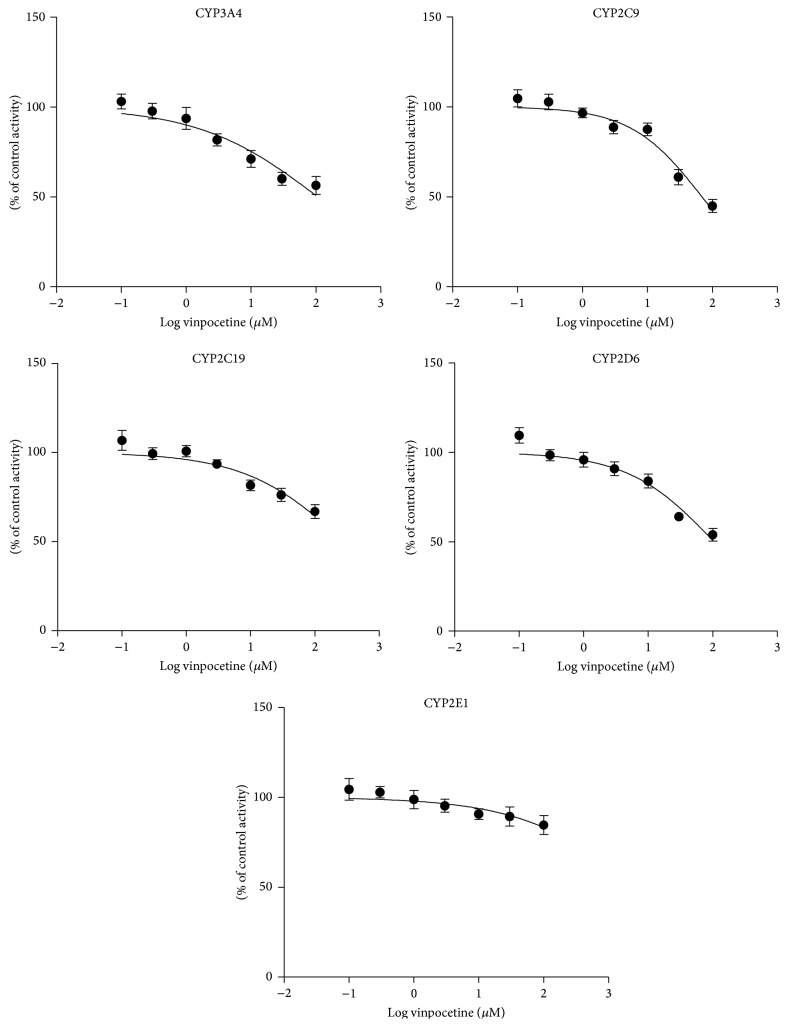
Inhibition curves of vinpocetine on five major CYP isoforms in human liver microsomes. Each data point represents the mean value (±SD) of triplicate determinations.

The IC_50_ values for five CYPs in human liver microsomes are presented in Table 1. The metabolite formations of each substrate (% of control) at the different dosages of vinpocetine are listed in Table 2. And the inhibition curves of vinpocetine on major cytochrome P450 isoforms in human liver microsomes are shown in Figure 2.

The results indicate that vinpocetine had a weak inhibitory effect on CYP2C9, with IC_50_ values of 68.91 *μ*M, whereas the IC_50_ values for CYP3A4, CYP2C19, CYP2D6, and CYP2E1 were all in excess of 100 *μ*M, indicated that vinpocetine did not affect CYP3A4, CYP2C19, CYP2D6, and CYP2E1 activities* in vivo*.

## 4. Discussions

Herbal medicines are often coadministered with therapeutic drugs, raising the potential of DDIs which are frequently caused by induction or inhibition of CYPs and/or P-gp [21, 22]. Up to the present, several publications have reported the interactions of vinpocetine with other clinical prescription drugs. According to Storm et al. [16], multiple doses of vinpocetine showed no influence on the steady state plasma concentrations and kinetics of oxazepam but cause diurnal changes in the plasma binding of oxazepam without clinical consequences. The values of AUC_0−*t*_, AUC_0−inf_, and *C*_max_ following the single administration of vinpocetine alone and after pretreatment of 5 days with omeprazole (10 mg/kg, intraperitoneally) were very similar in both groups [17]. According to a recent research result, vinpocetine induced minimal stimulation of the ATPase activity of the P-gp when compared to the positive controls verapamil [9].

However, there is a lack of information available regarding the metabolism mechanism of vinpocetine with additional drugs. Therefore, in this study we investigated the effects of vinpocetine on CYPs by using a cocktail method* in vitro*.

To examine the effects of vinpocetine on CYPs activity, a LC-MS/MS-based cocktail approach was used [20]. Previous studies documented that compounds with IC_50_ values higher than 100 *μ*M are believed to have no inhibitory effect because sufficiently high levels of these compounds are extremely unlikely to be achieved in the clinic, while IC_50_ values less than 10 *μ*M are considered potent inhibitors, and compounds with IC_50_ values between 10 and 50 *μ*M are considered moderate inhibitors [23, 24]. Our results (Tables 1 and 2, and Figure 2) indicated that vinpocetine showed weak inhibitory effect on the CYP2C9, where the IC_50_ value is 68.96 *μ*M; in addition, the IC_50_ values for CYP3A4, CYP2C19, CYP2D6, and CYP2E1 were all over range of 100 *μ*M, which showed that vinpocetine had almost no apparent inhibitory effects on these CYPs.

To the best of our knowledge, it is the first time to examine the effects of vinpocetine on CYPs activity* in vitro*. However, the needs are required to further* in vivo *study evaluation.

In conclusion, we clearly demonstrated the effect of vinpocetine on the activities of multiple CYP isoforms [19, 20]. It was found that vinpocetine showed weak inhibitory effect on the CYP2C9, whereas it had no apparent inhibitory effects on CYP3A4, CYP2C19, CYP2D6, and CYP2E1.

## Figures and Tables

**Table 1:** The IC_50_ values of vinpocetine on the activities of five major CYP isoenzymes in human liver microsomes.

Isoenzymes	Substrates	Metabolites	IC_50_ (*μ*M)
CYP3A4	Testosterone	6*β*-Hydroxytestosterone	107.1
CYP2C9	Tolbutamide	4-Hydroxytolbutamide	68.96 [95% CI: 49.14–96.79]
CYP2C19	Omeprazole	5-Hydroxyomeprazole	285.1
CYP2D6	Dextromethorphan	Dextrorphan	104.4
CYP2E1	Chlorzoxazone	6-Hydrxychlorzoxazone	2837

**Table 2:** Effects of vinpocetine on five major CYP-specific metabolite formations in human liver microsomes. Each data point represents the mean value (±SD) of triplicate determinations.

Isoenzymes (specific metabolites)	Metabolite formation (% of control)						
	Vinpocetine (*μ*g/mL)						
	0.1	0.3	1.0	3.0	10.0	30.0	100.0
CYP3A4 (6*β*-hydroxytestosterone)	103.1 ± 7.1	97.6 ± 7.5	93.6 ± 10.5	81.7 ± 5.8	71.1 ± 8.2	60.1 ± 6.2	56.4 ± 8.6
CYP2C9 (4-hydroxytolbutamide)	104.6 ± 8.3	102.7 ± 7.4	96.6 ± 4.6	88.7 ± 6.5	87.5 ± 6.0	60.9 ± 7.3	44.9 ± 6.2
CYP2C19 (5-hydroxyomeprazole)	106.7 ± 9.6	99.3 ± 5.7	100.5 ± 5.6	93.5 ± 4.0	81.5 ± 5.2	76.1 ± 6.4	66.8 ± 6.7
CYP2D6 (dextrorphan)	109.4 ± 7.4	98.3 ± 5.4	95.7 ± 7.1	90.7 ± 6.8	83.8 ± 6.7	64.0 ± 3.9	53.8 ± 6.1
CYP2E1 (6-hydrxychlorzoxazone)	104.4 ± 10.5	102.8 ± 5.4	98.8 ± 8.9	95.2 ± 6.2	90.6 ± 5.2	89.2 ± 9.2	84.5 ± 9.1
